# Illustrations of Heredity; Influence of Both Parents on the Children

**Published:** 1876-08

**Authors:** Ransom Dexter


### ILLUSTRATIONS OF HEREDITY; INFLUENCE OF BOTH PARENTS ON THE CHILDREN.

In calling the attention of the medical profession to a few remarkable cases of the pernicious influence of the use of alcoholic liquors by parents upon the minds and bodies of their offspring, it may not be out of place to indicate in the premises the factors through the action of which such conditions may be transmitted. It would be superfluous to bring forward evidence to show the hereditary tendencies in the constitution of both the body and mind under normal laws and circumstances, these facts having long since been settled by physiologists. The questions, however, relating to the hereditary transmission of temporary or acquired parental peculiarities, are not as fully recognized as they should be.

The following appear to me to be the essential conditions under which parental characteristics may be expected to be transmitted :

1. The natural healthy constitutions of father, mother, and their ancestors, are blended in their offspring.

2. The offspring of healthy individuals are likely to inherit the cast of the more strongly constituted parent, or the particular organs most highly developed, as they may appear in either of them.

3. In the acquired constitutional change, whether of disease or development, the impression may be so strongly made that the tendency to reproduce it again is stronger than to reassume the normal condition.

4. Strong mental impressions of either parent, though of comparatively short duration, may prove to be dominant in the offspring.

5. There may be a duality, in some instances, of mental impressions in the offspring on the same subject, having resulted from conflicting influences in the minds of the parents, as the following cases will serve to illustrate.

6. That parties whose ancestors have been drunkards, and who are constitutionally affected from that influence, show it in the lineaments of their bodies as well as in their mental peculiarities.

Through the consent of a grandchild of E. T., I have permission to report to the medical profession the following piece of family history, illustrating the baneful influence of alcoholic excesses, transmitted to the second, third, and fourth generations.

Case I. E. T., the first link in the chain of descent we describe, was a wealthy lumberman, descended from a highly respectable family, in which we have no account of any pre-existing nervous or mental disorder. Notwithstanding the fact that the habit of drinking was much more general among the better classes in our country sixty or seventy years ago than it is at present, there is no evidence to prove that he was, to any extent at least, the victim of heredity. Genteel, affable, and kind in his manners, he married at an early age, and commenced business with a fair prospect of success. Under the influence of associations, however, in the course of a few years he became confirmed in drinking habits, which in his case took on a periodic character, about two weeks being spent in a state of intoxition, alternating with about the same time in which he would drink but moderately. When intoxicated he appeared to possess a mania to abuse his family. If possible, it was a greater luxury to him than the ues of the alcoholic liquors he drank. With his mind stimulated to such an unnatural strain, and he striving continuously to render his abuse, by every means he could dedvise, as intense as possible, he became an object of horror at these times, to his wife and all others with whom he chanced to meet. He was so violent at times that it became necessary for all with whom he lived to leave their home and seek other quarters.

This state of their domestic relations remained as described five or six years, when a son, unfortunately for many others, was brought into existence. This son, J., early in life manifested many very peculiar eccentricities. While but a lad he would study to annoy his mother and other associates, devising every plan at his command to make trouble; but if it passed unnoticed, he would then exhibit his intention by violent abuse. But when he attained the age at which his father began to drink excessively, he then manifested every trait of character possessed by his father after he had become a drunkard, with one exception, namely, that he had his mother's aversion, which was exceedingly strong, to the use in any way whatever of alcoholic liquors. This son would not touch a drop of spirituous liquors, and would fairly detest any person who would, and yet he could not avoid his two weeks in every month of *mania* to abuse and annoy his family, which was carried to as great an extent, and in the same way as his father, but without ever having tasted a drop of alcohol. His family consisted of a wife and eight children, two of whom died while quite young; of the remaining six, but one possesses the father's habits, and that one has them in evry particular. In this one the horrid and detestable traits of character crop out as in the grandfather a half century ago.

The fourth grandchild in this family history has a son four years old, in whom both the mental and physical characteristics of his great-grandfather, after he had become an inebriate, are brought out in every particular.

Of the six adult grandchildren, the inebriate traits of character are impressed upon only one. There are now nine great-grandchildren, and only one well-marked case of this peculiar heredity.

Case II. N. J., the subject of the following peculiar history has attained the age of about forty; both he an his ancestors were healthy, industrious and intelligent.

Although alcoholic beverages were used quite sparingly by his parents, the mother when *enciente* was in the habit of using them several times daily until after confinement. But just before becoming pregnant with the subject of these remarks, her eighth child, a strong temperance movement was instituted in the section of the country in which his parents resided. His father being a minister, was naturally expected to take the lead in the good cause. Both parents taking an active part in the endeavor to suppress the use of alcoholic liquors, of course forbade the accustomed use of the beverage even in the delicate situation in which she had now become. During the latter months of gestation a great conflict ensued between the appetite for the beverage to which she had been accustomed, and the moral restraint to which she had subjected herself. The appetite growing stronger as gestation advanced, was a source of great discomfort; but the moral restraint and the power of the will held the entire mastery.

The effect upon the offspring was specific. The boy when quite small was often the object of observation. Quite to the surprise of his parents he manifefted the same peculiarities of appetite and sentiment that possessed his mother during her stage of gestation with him. If there was any spirituous liquor anywhere near him he would be frantic to get a drink of it, but strange as it may seem, would appear glad when it was placed beyond his reach.

This gentleman reports, and his veracity is perfectly reliable, that from his earliest recollection he has always had a strong appetite and desire for alcoholic liquors, but is cognizant of the accompanying automatic action of the will, which enables him to abstain from them. He says that he has a conscious sensation within his own mind that the desire for these liquors is coupled, in the mind's action, with the will-power that enables him to abstain from their use. In short, he has a duality in the operations of his mind transmitted from his mother, and exactly as she had them.—*Ransom Dexter, A.M., M.D., in Chicago Journal.*

